# Comparison of Ovarian-Adnexal Reporting and Data System (O-RADS) Ultrasound and International Ovarian Tumor Analysis (IOTA) Simple Rules in Characterizing Benign and Malignant Ovarian Lesions: A Retrospective Study

**DOI:** 10.7759/cureus.105757

**Published:** 2026-03-24

**Authors:** Suprith J Shankar, Anil K Sakalecha, Goravimakalahalli Srinivasareddy Hemanth Kumar, Kalyani Raju, Soumya Chincholikar

**Affiliations:** 1 Radiodiagnosis, Sri Devaraj Urs Medical College, Kolar, IND; 2 Pathology, Sri Devaraj Urs Medical College, Kolar, IND

**Keywords:** adnexal masses, iota simple rules, o-rads ultrasound, ovarian neoplasms, ultrasound

## Abstract

Objective

The objective of this study is to compare the diagnostic accuracy of the Ovarian-Adnexal Reporting and Data System (O-RADS) and the International Ovarian Tumor Analysis (IOTA) Simple Rules in distinguishing benign from malignant ovarian masses, using histopathology as the reference standard.

Methods

This study was a retrospective observational investigation carried out in a tertiary care facility and included women with ovarian masses who underwent ultrasound evaluation between January 2023 and December 2025, followed by surgical excision with histopathological confirmation. Ultrasound images were retrospectively reviewed by two radiologists who were blinded to the final histopathological diagnosis. In cases of disagreement, a consensus decision was reached. The lesions were classified according to O-RADS and IOTA Simple Rules based on the recorded imaging features. O-RADS categories 4-5 were considered positive for malignancy. Inconclusive IOTA cases were excluded from performance analysis. Sensitivity, specificity, positive predictive value (PPV), negative predictive value (NPV), and accuracy were calculated. Multivariate logistic regression was performed to identify independent predictors of malignancy.

Results

Histopathology confirmed 24 (30%) malignant and 56 (70%) benign lesions. O-RADS demonstrated 91.7% sensitivity, 82.1% specificity, 68.8% PPV, 95.8% NPV, and 85.0% overall accuracy. IOTA yielded 12 (15%) inconclusive cases. After exclusion, IOTA showed 91.7% sensitivity, 95.5% specificity, 91.7% PPV, 95.5% NPV, and 94.1% accuracy. In a secondary analysis considering inconclusive cases as malignant, IOTA demonstrated 92.3% sensitivity, 78.6% specificity, 66.7% PPV, 95.7% NPV, and 85% accuracy. Solid components (OR 8.4), very strong vascularity (OR 7.5), and ascites (OR 6.9) were independent predictors of malignancy.

Conclusions

Both O-RADS and IOTA Simple Rules demonstrate high diagnostic performance in distinguishing benign from malignant adnexal lesions. O-RADS offers greater sensitivity and applicability across all lesions, supporting its role in clinical triage, while IOTA provides higher specificity in classifiable cases but is limited by inconclusive results. When such cases are included, diagnostic performance becomes more comparable. O-RADS, therefore, represents a more consistent and clinically applicable framework.

## Introduction

Adnexal masses frequently occur across a wide age range of women and commonly prompt the need for pelvic imaging. Although most adnexal lesions are benign, ovarian malignancy significantly contributes to gynecologic cancer mortality, primarily due to delayed diagnosis [1,2]. Accurate preoperative characterization of ovarian masses is essential to guide clinical management, prevent unnecessary surgical procedures, and facilitate timely oncologic referral when indicated [2].

Ultrasound is the first-line imaging modality for ovarian mass evaluation because of its accessibility, real-time imaging, and absence of ionizing radiation [3]. Variability in descriptive terminology and subjective interpretation has historically reduced reproducibility and interobserver agreement [4].

Structured risk stratification systems have been developed to enhance standardization. The International Ovarian Tumor Analysis (IOTA) group introduced Simple Rules, classifying lesions according to predefined benign and malignant ultrasound features [5]. Further multicenter validation studies confirmed the diagnostic robustness of the IOTA framework [6,7]. More recently, the American College of Radiology introduced the Ovarian-Adnexal Reporting and Data System (O-RADS), incorporating a standardized lexicon and assigning malignancy risk categories to enhance reporting uniformity and clinical communication [8]. Validation studies demonstrated good diagnostic performance of O-RADS in adnexal mass classification [9,10]. The 2022 update refined risk stratification and management recommendations [11].

Despite widespread adoption, comparative validation of these systems in real-world clinical settings remains essential [12-14]. This study compares the diagnostic performance of O-RADS and IOTA Simple Rules in distinguishing benign from malignant ovarian masses, using histopathology as the gold standard in a retrospective observational cohort.

## Materials and methods

Study design and population

This retrospective observational study was conducted at R L Jalappa Hospital & Research Center, a tertiary care center affiliated with Sri Devaraj Urs Medical College, Kolar, India, following approval by the institutional ethics committee. Because the study was retrospective in nature, the informed consent requirement was waived.

Women with adnexal masses who underwent ultrasound evaluation between January 2023 and December 2025, followed by surgical excision and histopathological confirmation, were identified through hospital records and imaging archives.

Inclusion and exclusion criteria

Adult women who had a documented preoperative transvaginal or transabdominal ultrasound examination and a definitive histopathological diagnosis of the adnexal lesion were included. Patients were excluded if they had incomplete imaging records or inadequate image quality that prevented retrospective classification according to O-RADS or IOTA criteria; a history of prior pelvic or ovarian surgery; chemotherapy or radiation for pelvic or adnexal malignancies; or non-ovarian adnexal lesions (e.g., hydrosalpinx, paraovarian cysts, ectopic pregnancy, and subserosal fibroid).

**Figure 1:**
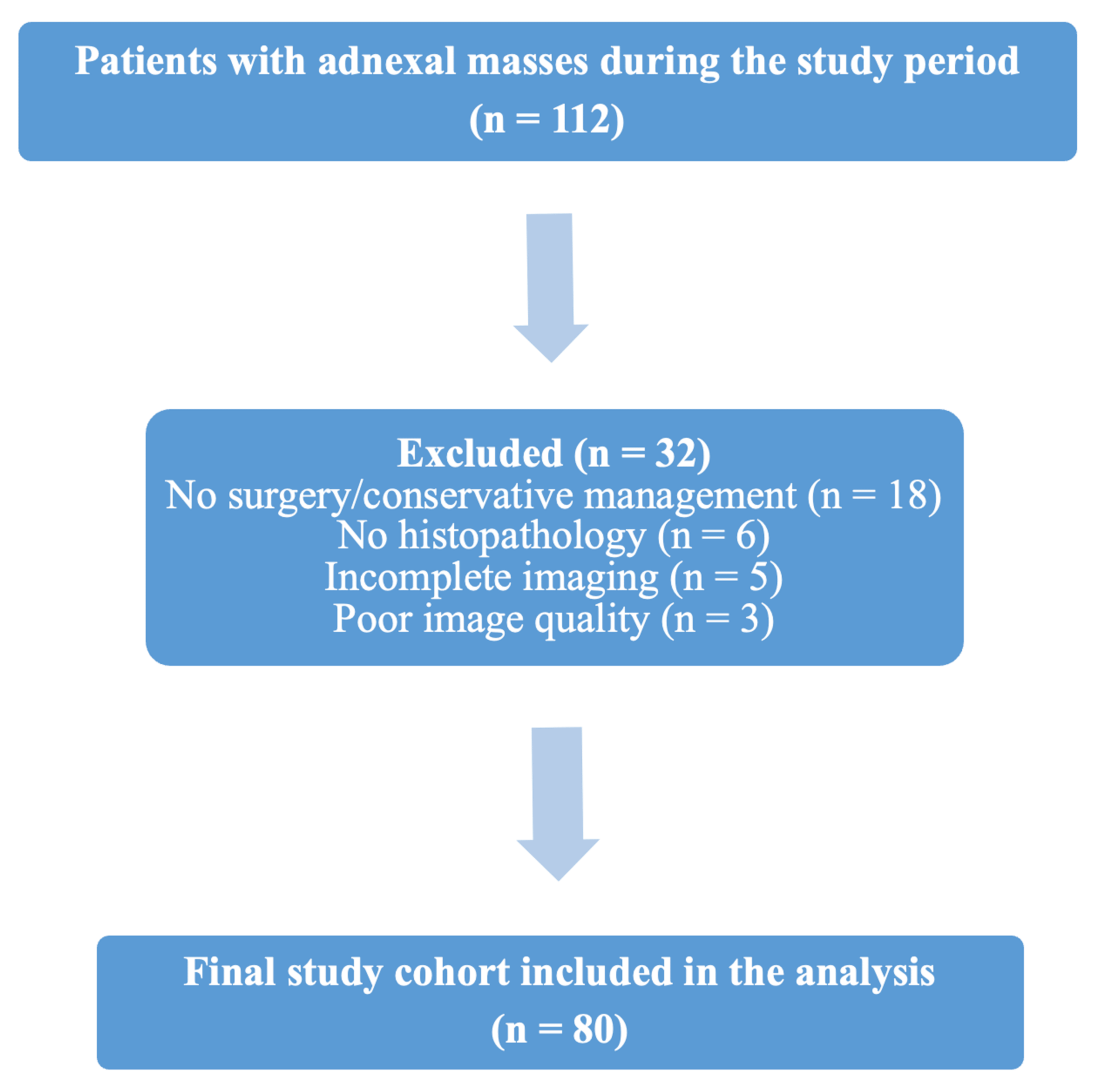
Flow diagram illustrating selection of patients and final study cohort

A non-probabilistic consecutive sampling method was employed to include all eligible patients during the study period. Initially, 112 patients with adnexal masses were identified between January 2023 and December 2025. After applying the predefined inclusion and exclusion criteria, 80 patients comprised the final study cohort. The process of patient selection is presented in Figure 1.

Ultrasound evaluation

Ultrasound examinations were conducted using high-resolution transvaginal or transabdominal probes during routine clinical care. Two radiologists, blinded to the final histopathological diagnosis, independently and retrospectively reviewed the ultrasound images, including available static images and cine loops. Lesions were assessed based on recorded morphological features and Doppler findings. Cases with discrepancies were jointly reevaluated, and a final interpretation was reached by consensus. Documented lesion characteristics included morphology, septations, papillary projections, wall irregularity, solid components, and vascularity.

O-RADS classification

O-RADS categorization was assigned according to the American College of Radiology O-RADS ultrasound risk stratification system [8] based on recorded imaging findings. For diagnostic performance analysis, categories 0-3 were classified as negative for malignancy, while categories 4 and 5 were classified as positive.

IOTA Simple Rules

The IOTA Simple Rules were applied retrospectively using documented ultrasound features according to the criteria established by the IOTA group [5]. Lesions were classified as benign, malignant, or inconclusive based on predefined benign and malignant features. Inconclusive cases were excluded from the primary sensitivity and specificity analyses, as the IOTA Simple Rules are intended to classify lesions only when definitive features are present. Additionally, a secondary analysis was performed by considering inconclusive cases as malignant to allow an intent-to-diagnose comparison with O-RADS.

Histopathological analysis

Histopathological examination of surgical specimens was used as the diagnostic reference standard. Pathologists with more than 10 years of experience in gynecologic pathology evaluated all resected ovarian lesions using standard histopathological techniques. In routine practice, difficult or equivocal cases were reviewed in consensus among pathologists to reach a final diagnosis. Final diagnoses were obtained from pathology reports, and lesions were classified as benign or malignant.

Statistical analysis

Data analysis was performed using IBM SPSS Statistics for Windows, Version 25.0 (Released 2017; IBM Corp., Armonk, NY, USA). Categorical variables were reported as frequencies and percentages and compared using Fisher’s exact test or the chi-square test. The assumptions of the chi-square test, including independence of observations and adequate expected cell frequencies, were assessed prior to analysis, and Fisher’s exact test was used when expected cell counts were less than 5. Continuous variables were reported as mean ± SD. Diagnostic performance metrics, including sensitivity, specificity, positive predictive value (PPV), negative predictive value (NPV), and accuracy, were calculated with histopathology serving as the reference standard. Multivariate logistic regression was performed to identify independent predictors of malignancy. A p-value less than 0.05 was considered statistically significant.

## Results

The study included 80 women with adnexal masses, selected using a non-probabilistic consecutive sampling approach. Histopathological examination identified 24 (30%) malignant lesions and 56 (70%) benign lesions. The mean patient age was 44.2 ± 11.6 years (range, 21-68 years). Malignancy occurred more frequently in postmenopausal women (n = 14, 58.3%) than in premenopausal women (n = 10, 41.7%).

Benign lesions comprised serous cystadenoma, mucinous cystadenoma, mature cystic teratoma, endometrioma, and fibroma. Malignant lesions included serous carcinoma, clear cell carcinoma, endometrioid carcinoma, and mucinous carcinoma. Among benign lesions, serous cystadenoma (n = 18) and mature cystic teratoma (n = 12) were most common. Malignant lesions were predominantly serous carcinomas (n = 10), followed by mucinous and endometrioid carcinomas.

Table 1 summarizes the baseline clinicopathological characteristics of the study population, including age distribution, menopausal status, and histopathological diagnosis.

**Table 1:** Baseline clinicopathological characteristics of the study population (n = 80) Values are presented as numbers (percentages) unless otherwise specified. The mean age is expressed as mean ± SD.

Characteristic	n (%)/Value
Demographic characteristics	
Total patients	80
Mean age (years)	44.2 ± 11.6
Premenopausal	52 (65%)
Postmenopausal	28 (35%)
Lesion classification	
Benign lesions	56 (70%)
Malignant lesions	24 (30%)
Histopathology - benign lesions	
Serous cystadenoma	18 (22.5%)
Mucinous cystadenoma	10 (12.5%)
Mature cystic teratoma	12 (15%)
Endometrioma	9 (11.3%)
Fibroma	7 (8.7%)
Histopathology - malignant lesions	
Serous carcinoma	10 (12.5%)
Mucinous carcinoma	5 (6.3%)
Endometrioid carcinoma	5 (6.3%)
Clear cell carcinoma	4 (5%)

Ultrasound morphological characteristics

Categorical variables between benign and malignant ovarian lesions were compared using the chi-square test or Fisher’s exact test, as appropriate. The assumptions of the chi-square test, including independence of observations and adequate expected cell frequencies, were assessed prior to analysis, and Fisher’s exact test was applied for variables with small expected cell counts.

Ultrasound evaluation revealed that malignant lesions were significantly associated with solid components, irregular walls, papillary projections, increased vascularity, thick septations, and ascites. Solid components were present in 20 (83%) of malignant lesions compared to 12 (21%) of benign lesions (p < 0.001). High vascularity (color score 3-4) was observed in 19 (79%) of malignant masses versus nine (16%) of benign masses (p < 0.001). Ascites was detected in 14 (58%) of malignant cases compared to three (5%) of benign cases (p < 0.001).

Table 2 presents the distribution of ultrasound morphological characteristics in benign and malignant ovarian lesions.

**Table 2:** Distribution of ultrasound morphological characteristics in benign and malignant ovarian lesions Values are expressed as numbers (percentages). Categorical variables were compared using the chi-square test after verifying the test assumptions. A p-value < 0.05 was considered statistically significant. A color score of 3-4 indicates high vascularity.

Feature	Benign (n = 56)	Malignant (n = 24)	χ² Value	p-Value
Solid component	12 (21%)	20 (83%)	26.83	<0.001
Papillary projections	8 (14%)	18 (75%)	28.23	<0.001
Thick septations (>3 mm)	10 (18%)	16 (67%)	18.24	<0.001
Multiloculated cystic lesion	22 (39%)	12 (50%)	0.79	0.374
Irregular wall	6 (11%)	17 (71%)	29.64	<0.001
High vascularity (color score 3-4)	9 (16%)	19 (79%)	29.4	<0.001
Ascites	3 (5%)	14 (58%)	28.17	<0.001

O-RADS distribution

The likelihood of malignancy increased progressively with higher O-RADS categories. Lesions classified as O-RADS 2 were exclusively benign, while the majority of O-RADS 5 lesions were malignant. Table 3 presents the distribution of benign and malignant lesions across O-RADS categories.

**Table 3:** Distribution of benign and malignant lesions across O-RADS ultrasound categories O-RADS, Ovarian-Adnexal Reporting and Data System

O-RADS category	Benign (n = 56)	Malignant (n = 24)	Total
O-RADS 2	18	0	18
O-RADS 3	28	2	30
O-RADS 4	8	10	18
O-RADS 5	2	12	14
Total	56	24	80

IOTA distribution

The application of the IOTA Simple Rules facilitated the characterization of adnexal masses using predefined benign and malignant ultrasound features. Twelve (15%) cases were classified as inconclusive according to the IOTA criteria. Table 4 summarizes the distribution of benign and malignant ultrasound features alongside the final IOTA classification.

**Table 4:** Distribution of benign and malignant ultrasound features according to the IOTA Simple Rules and final classification of ovarian lesions Categorical variables were analyzed using Fisher’s exact test due to small cell counts in several categories. A p-value < 0.05 was considered statistically significant. IOTA, International Ovarian Tumor Analysis

Parameter	Benign (n = 56)	Malignant (n = 24)	Total	p-Value
Benign features				
Unilocular cyst	20 (35.7%)	0	20	<0.001
Acoustic shadows	16 (28.6%)	0	16	<0.001
Smooth multilocular <100 mm	12 (21.4%)	1 (4.2%)	13	0.11
No blood flow	18 (32.1%)	1 (4.2%)	19	0.01
Malignant features				
Irregular solid tumor	2 (3.6%)	17 (70.8%)	19	<0.001
≥4 papillary projections	1 (1.8%)	11 (45.8%)	12	<0.001
Irregular multilocular-solid >100 mm	3 (5.4%)	13 (54.2%)	16	<0.001
Ascites	3 (5.4%)	14 (58.3%)	17	<0.001
Very strong blood flow	4 (7.1%)	15 (62.5%)	19	<0.001
Final IOTA classification				
Benign	44	2	46	-
Malignant	2	22	24	-
Inconclusive	10	2	12	-

**Figure 2:**
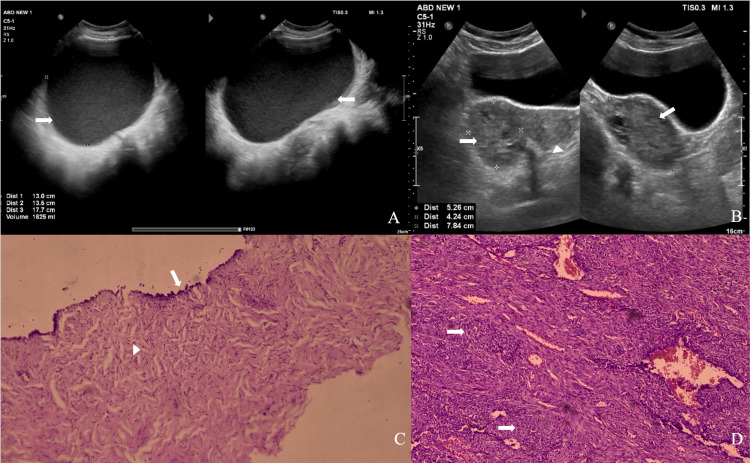
Representative ultrasound and histopathological correlation of ovarian lesions (A) Ultrasound grayscale image showing a well-defined anechoic cystic lesion (arrow) with internal echoes in the right adnexa, suggestive of a benign ovarian cyst (O-RADS 2; IOTA benign). (B) Ultrasound grayscale image showing a fairly defined heterogeneous solid lesion in the right adnexa (arrow) with a few internal cystic areas, suggestive of a malignant ovarian lesion (O-RADS 4; IOTA malignant). Arrowhead indicates the uterus. (C) Histopathological photomicrograph demonstrating lining by a single layer of tall columnar epithelial cells (arrow) with underlying fibrous stroma containing spindle fibroblasts (arrowhead), consistent with serous cystadenoma (H&E stain, ×200). (D) Histopathological photomicrograph demonstrating stroma replaced by pleomorphic high-grade neoplastic cells arranged in clusters and sheets (arrow), consistent with serous cystadenocarcinoma (H&E stain, ×200). O-RADS, Ovarian-Adnexal Reporting and Data System

Representative ultrasound images of benign and malignant adnexal lesions, along with corresponding histopathological correlations, are shown in Figure 2.

Diagnostic performance comparison

Using O-RADS ≥ 4 as the malignancy threshold, O-RADS demonstrated 91.7% sensitivity, 82.1% specificity, 68.8% PPV, 95.8% NPV, and 85.0% overall diagnostic accuracy. After excluding inconclusive cases, the IOTA model demonstrated 91.7% sensitivity, 95.5% specificity, 91.7% PPV, 95.5% NPV, and 94.1% overall accuracy.

To address potential selection bias due to exclusion of inconclusive cases, an additional analysis was performed by treating inconclusive IOTA cases as malignant. In this analysis, IOTA demonstrated 92.3% sensitivity, 78.6% specificity, 66.7% PPV, 95.7% NPV, and 85.0% overall accuracy, reflecting a more conservative, intent-to-diagnose approach.

The diagnostic performance of O-RADS and IOTA Simple Rules in distinguishing benign from malignant ovarian lesions, using histopathology as the gold standard, is presented in Table 5.

**Table 5:** Diagnostic performance of O-RADS and IOTA Simple Rules for differentiation of benign and malignant ovarian lesions, with IOTA results shown for both primary and secondary analyses IOTA, International Ovarian Tumor Analysis; NPV, negative predictive value; O-RADS, Ovarian-Adnexal Reporting and Data System; PPV, positive predictive value

Parameter	O-RADS	IOTA (primary analysis: excluding inconclusive cases)	IOTA (secondary analysis: including inconclusive cases as malignant)
Sensitivity	91.70%	91.70%	92.30%
Specificity	82.10%	95.50%	78.60%
PPV	68.80%	91.70%	66.70%
NPV	95.80%	95.50%	95.70%
Accuracy	85.00%	94.10%	85.00%

Multivariate logistic regression analysis of significant ultrasound variables identified the presence of a solid component (OR 8.4; 95% CI, 2.3-30.7; p = 0.001), very strong vascularity (OR 7.5; 95% CI, 2.1-27.4; p = 0.002), and ascites (OR 6.9; 95% CI, 1.8-26.1; p = 0.004) as independent predictors of malignancy. Table 6 presents the independent ultrasound predictors of malignancy identified through multivariate logistic regression analysis.

**Table 6:** Multivariate logistic regression analysis showing independent ultrasound predictors of malignancy in ovarian masses A p-value < 0.05 was considered statistically significant.

Variable	OR	95% CI	p-Value
Papillary projections	2.1	0.6-7.4	0.23
Thick septations	1.8	0.5-6.3	0.34
Solid component	8.4	2.3-30.7	0.001
Very strong vascularity	7.5	2.1-27.4	0.002
Ascites	6.9	1.8-26.1	0.004

## Discussion

In this retrospective observational cohort study, both O-RADS and IOTA Simple Rules exhibited high diagnostic performance in distinguishing benign from malignant ovarian lesions. O-RADS achieved 91.7% sensitivity and 95.8% NPV, whereas IOTA Simple Rules demonstrated a specificity of 95.5% and an overall accuracy of 94.1% after excluding inconclusive cases. When inconclusive cases were considered malignant in a secondary analysis, sensitivity increased to 92.3% while specificity decreased to 78.6%, with an overall accuracy of 85.0%. This reflects a more conservative, intent-to-diagnose approach and highlights the impact of inconclusive cases on IOTA performance.

The sensitivity observed for O-RADS in our study is comparable to that reported by Basha et al. [12], who demonstrated a sensitivity of 96% for O-RADS in identifying malignant adnexal masses. In their comparative study of O-RADS and IOTA systems, specificity for O-RADS was approximately 80%, similar to the 82.1% specificity observed in our cohort. The moderate specificity in both studies reflects the classification of certain complex benign lesions into higher-risk categories, prioritizing oncologic safety.

In our study, no malignant lesions were categorized as O-RADS 2, and only two malignant lesions were assigned to O-RADS 3. This distribution is consistent with risk stratification reported by the American College of Radiology O-RADS committee [8], in which O-RADS 2 lesions demonstrate a malignancy risk of <1% and O-RADS 3 lesions demonstrate a malignancy risk of <10%.

The performance of IOTA Simple Rules in our cohort is consistent with the original validation study by Timmerman et al. [5], which reported 93% sensitivity and 90% specificity in classifiable lesions. In our study, IOTA achieved 91.7% sensitivity and 95.5% specificity after excluding inconclusive cases. The inconclusive rate of 15% in our study is within the range reported by Timmerman et al. [5], in which approximately 15-20% of cases were not classifiable using Simple Rules alone.

Almeida et al. [13], in a systematic review and meta-analysis evaluating IOTA Simple Rules, O-RADS, and the ADNEX model, reported pooled sensitivity of approximately 91% and specificity of 87% for IOTA Simple Rules. Similarly, the pooled sensitivity for O-RADS in their analysis exceeded 90%, which aligns closely with our results.

Multivariate logistic regression identified solid components (OR 8.4), strong vascularity (OR 7.5), and ascites (OR 6.9) as independent predictors of malignancy. These findings agree with the IOTA group’s original observations [5] that irregular solid tumors and increased color Doppler flow are among the strongest predictors of malignancy. Ascites has also been described as a high-risk feature in both O-RADS and IOTA frameworks [5,8].

Clinically, O-RADS offers a structured lexicon and risk categories for all lesions, improving standardization and communication [8]. IOTA Simple Rules provide higher specificity but can produce inconclusive classifications that require further assessment [5]. This difference may explain the slightly higher specificity of IOTA observed in both our study cohort and prior comparative analyses [12-14].

This study is strengthened by several key features. It provides a direct comparison of O-RADS and IOTA Simple Rules within the same patient cohort using histopathology as the reference standard. The inclusion of both primary and secondary analyses for IOTA allows a more comprehensive and clinically relevant evaluation. Additionally, the use of standardized imaging criteria and consensus review by experienced radiologists enhances the reliability of the imaging assessment.

Limitations of this study include the moderate sample size, retrospective design, and inclusion of only surgically treated lesions with histopathological confirmation, which may introduce selection bias and limit generalizability. Additionally, exclusion of inconclusive IOTA cases in the primary analysis may introduce selection bias and overestimate diagnostic performance, as results apply only to classifiable lesions; a secondary analysis including these cases was performed to address this limitation.

Another limitation is the retrospective assessment of Doppler vascularity from stored images. As O-RADS and IOTA grading are ideally performed in real time, evaluation using static images or limited cine loops may lead to misclassification. Efforts were made to minimize this by reviewing multiple image frames and cine loops with consensus reading by two radiologists.

## Conclusions

This retrospective observational study found that both O-RADS and IOTA Simple Rules demonstrated high diagnostic performance in distinguishing benign from malignant ovarian lesions. O-RADS showed excellent sensitivity and provides comprehensive risk stratification applicable to all lesions, supporting its role as an effective tool for screening and clinical triage. In contrast, IOTA Simple Rules demonstrated higher specificity when lesions were classifiable; however, inconclusive cases limit its applicability and may lead to overestimation of diagnostic performance when excluded. When such cases are incorporated, diagnostic performance becomes more comparable to O-RADS. Overall, O-RADS offers a more consistent and clinically applicable framework, while IOTA remains useful for improving specificity in clearly classifiable cases. Prospective multicenter studies are needed to further validate and refine the comparative use of these classification systems.
